# Recurrent spreading depolarizations after subarachnoid hemorrhage decreases oxygen availability in human cerebral cortex

**DOI:** 10.1002/ana.21943

**Published:** 2010-05

**Authors:** Bert Bosche, Rudolf Graf, Ralf-Ingo Ernestus, Christian Dohmen, Thomas Reithmeier, Gerrit Brinker, Anthony J Strong, Jens P Dreier, Johannes Woitzik, Members of the Cooperative Study of Brain Injury Depolarizations (COSBID)

**Affiliations:** 1 Department of Neurosurgery, University of CologneCologne, Germany; 2 Max-Planck-Institute for Neurological ResearchCologne, Germany; 3 Department of Psychiatry, University of DüsseldorfDüsseldorf, Germany; 4 Department of Neurosurgery, University of WürzburgWürzburg, Germany; 5 Department of Neurology, University of CologneCologne, Germany; 6 Department of Stereotactical Neurosurgery, Neurocenter, University of FreiburgFreiburg, Germany; 7 Department of Clinical Neuroscience, King's College London, Institute of PsychiatryLondon, UK; 8 Departments of Neurology and Experimental Neurology, Charité Campus Mitte, University Medicine BerlinBerlin, Germany; 9 Center of Stroke ResearchBerlin, Germany; 10 Department of Neurosurgery, University Medicine MannheimMannheim, Germany; 11 Department of Neurosurgery, Charité Campus Benjamin Franklin, University Medicine BerlinBerlin, Germany

## Abstract

**Objective:**

Delayed ischemic neurological deficit (DIND) contributes to poor outcome in subarachnoid hemorrhage (SAH) patients. Because there is continuing uncertainty as to whether proximal cerebral artery vasospasm is the only cause of DIND, other processes should be considered. A potential candidate is cortical spreading depolarization (CSD)-induced hypoxia. We hypothesized that recurrent CSDs influence cortical oxygen availability.

**Methods:**

Centers in the Cooperative Study of Brain Injury Depolarizations (COSBID) recruited 9 patients with severe SAH, who underwent open neurosurgery. We used simultaneous, colocalized recordings of electrocorticography and tissue oxygen pressure (p_ti_O_2_) in human cerebral cortex. We screened for delayed cortical infarcts by using sequential brain imaging and investigated cerebral vasospasm by angiography or time-of-flight magnetic resonance imaging.

**Results:**

In a total recording time of 850 hours, 120 CSDs were found in 8 of 9 patients. Fifty-five CSDs (∼46%) were found in only 2 of 9 patients, who later developed DIND. Eighty-nine (∼75%) of all CSDs occurred between the 5th and 7th day after SAH, and 96 (80%) arose within temporal clusters of recurrent CSD. Clusters of CSD occurred simultaneously, with mainly biphasic CSD-associated p_ti_O_2_ responses comprising a primary hypoxic and a secondary hyperoxic phase. The frequency of CSD correlated positively with the duration of the hypoxic phase and negatively with that of the hyperoxic phase. Hypoxic phases significantly increased stepwise within CSD clusters; particularly in DIND patients, biphasic p_ti_O_2_ responses changed to monophasic p_ti_O_2_ decreases within these clusters. Monophasic hypoxic p_ti_O_2_ responses to CSD were found predominantly in DIND patients.

**Interpretation:**

We attribute these clinical p_ti_O_2_ findings mainly to changes in local blood flow in the cortical microcirculation but also to augmented metabolism. Besides classical contributors like proximal cerebral vasospasm, CSD clusters may reduce O_2_ supply and increase O_2_ consumption, and thereby promote DIND. ANN NEUROL 2010;67:607–617

Cerebral vasospasm of large proximal arteries is regarded as the prime cause of delayed ischemic neurological deficit (DIND) after subarachnoid hemorrhage (SAH). It is, however, only 1 possible contributor to DIND.^1^,^2^ Thus, a search is required for alternative or additional mechanisms to explain delayed deterioration after SAH.^1^,^3^ DIND has recently been associated with cortical spreading depolarization (CSD),^4^ a striking electrophysiological phenomenon that was originally described as “cortical spreading depression” by Leão in 1944.^5^,^6^ CSD is a self-propagating wave of neuronal and glial depolarization that has been extensively investigated in animals.^6^–^8^ A series of recent clinical studies using subdural electrocorticography (ECoG) filtered to include low frequencies provides direct and unequivocal electrophysiological evidence for the existence of CSDs in neurotrauma,^9^ malignant middle cerebral artery infarction,^10^ and SAH.^4^ These studies suggest that CSD may be a general phenomenon leading to secondary deterioration in various clinical entities.^11^

CSD waves are accompanied by propagating failure of brain ion homeostasis resulting in an interruption of cortical function.^7^ Under physiological conditions, the brain is able fully to recover from the metabolic challenge of reestablishing ion homeostasis during the repolarization phase of CSD without measurable tissue damage.^12^ This ability is mainly achieved by a compensatory vasodilatory response coupled to CSD. In contrast, recurrent CSDs emerging spontaneously in metabolically compromised tissue, such as in the ischemic penumbra (in this situation termed peri-infarct depolarizations, [PIDs])^13^ or after SAH,^4^ irreversibly damage brain tissue at risk and thereby may lead to lesion growth.^10^,^14^,^15^ Animal experiments have suggested that, under such conditions of impaired metabolic status, the vasodilatory response to CSD switches from a compensatory vasodilatory response to an inverse, vasoconstrictive neurovascular coupling, resulting in cortical spreading ischemia (CSI).^16^–^19^ In a rat model, it has been hypothesized that, in principle, CSD activates both vasoconstriction and vasodilatation in a biphasic fashion, the ratio between the 2 being shifted toward vasoconstriction by ischemic/hypoxic tissue conditions.^20^ Recently, combined recordings of regional cerebral blood flow (CBF) and ECoG using subdural optoelectrode strips have provided evidence that delayed ischemic stroke is associated with CSI in patients with SAH.^15^ To our knowledge, this is the first detailed report of continuously corecorded tissue oxygen pressure (p_ti_O_2_) and CSD in human cerebral cortex. We hypothesized that CSDs, in particular if they occur in temporal clusters, reduce cortical oxygen availability.

## Materials and Methods

The study was designed and performed as a substudy within the Cooperative Study of Brain Injury Depolarizations (COSBID) study group. Patients or their relatives gave written consent for study inclusion. The study was approved by the local ethics committees of the Universities of Cologne and Mannheim/Heidelberg. At these centers, 9 patients with acute SAH due to spontaneous rupture of an intracranial aneurysm were studied, where imaging^21^ and clinical^4^,^22^,^23^ characteristics indicated a high risk of DIND development. Patients were treated according to German guidelines for aneurysmal SAH (Association of the Scientific Medical Societies in Germany; see http://www.AWMF.org). If a low level of consciousness required it, patients were ventilated and sedated using midazolam + fentanyl/sufentanil (n = 8). Patient 3 was partly ventilated and sedated, and breathed spontaneously in the later part of the monitoring. When craniotomy was required either for aneurysm clipping, or for intracranial hemorrhage evacuation in patients in whom the aneurysm was coiled, electrode and p_ti_O_2_ probe implantations were performed before closure of the craniotomy, for later recording of these variables.

### Implantation of ECoG Strip Electrodes and p_ti_O_2_ Probes

**FIGURE 1:**
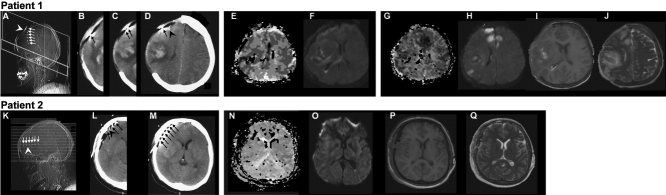
X-ray topographic images and cranial computed tomography (CT) slices of Patients 1 and 2 with individual locations of the electrocorticography (ECoG) strip electrodes (*arrows*, CT artifacts of electrodes) and the oxygen partial pressure probes (*arrowheads*) adjacent to strip electrodes. Please note that levels of axial sections differ somewhat among scans. The upper level shows Patient 1, with subarachnoid hemorrhage predominantly in the sylvian fissure with additional intraparenchymal hemorrhage and perifocal space-occupying edema. (A-D) A CT scan was performed after decompressive craniectomy with ECoG and tissue oxygen pressure (p_ti_O_2_) probe implantation 2 days after subarachnoid hemorrhage (SAH). (A) In the topogram, the arrows represent 6 single electrodes of the ECoG strip electrode. The arrowhead demarcates the p_ti_O_2_ probe. (B) The arrow indicates electrode 2. (C) Arrows indicate electrodes 3 and 4, respectively. (D) The arrow indicates electrode 5, and the arrowhead demarcates the p_ti_O_2_ probe. (E) Perfusion-weighted (PW) magnetic resonance imaging (MRI) and (F) diffusion-weighted (DW) MRI show no perfusion deficit or signs of cortical ischemia 4 days after SAH. The area of intracranial hemorrhage displays disturbance of the diffusion signal. (G) PW MRI and (H) DW MRI demonstrate areas of hypoperfusion and signs of cortical ischemia predominantly of the right frontal lobe, but also of the left frontal lobe 9 days after SAH. (I) MRI (T1 weighted) shows infarcted cortical tissue. (J) A T2-weighted MRI cross-section (more rostral) gives a detailed picture of the distribution of cortical infarcts with right frontoparietal and bifrontal representation. The lower level shows Patient 2, who suffered a subarachnoid hemorrhage from a ruptured aneurysm, which was clipped surgically 2 days after SAH. Cranial CT scan was performed 1 day after the craniotomy and ECoG and p_ti_O_2_ probe implantation. (K) X-ray topogram and (K-M) 2 different slices are depicted. (K) Six arrows indicate the ECoG strip with 6 single electrodes. The arrowhead demarcates the p_ti_O_2_ probe. (L) The arrows indicate electrodes 1, 2, and 3; the arrowhead indicates the p_ti_O_2_ probe between electrodes 2 and 3. (M) All 6 electrodes are displayed; however, the p_ti_O_2_ probe is not visible on this cross section. (N) Seven days after SAH, PW MRI showed no hypoperfusion, and (O) DW MR imaging revealed no signs of ischemia (disturbance of diffusion). (P) The same was true for the T1-weighted MRI and also (Q) for T2-weighted MRI (see Supplementary Clinical Results online for a more detailed description of the 2 individual clinical courses).

The ECoG strip electrode (Ad-Tech Medical, Racine, WI) was placed on the cerebral cortex as reported previously (Fig 1).^4^ In addition, a Clark-type probe for p_ti_O_2_ monitoring (Licox CC1-SB, Integra Neurosciences, Andover, UK) was inserted in the cerebral cortex. The 0.5mm thin probe was placed adjacent to the ECoG strip (≤8mm) between the single electrodes (see Fig 1D, L). It samples a tissue compartment of 7.1 to 15mm^2^.^24^ Neither bleeding nor infection was seen associated with placement or removal of either the ECoG electrode strip or the p_ti_O_2_ probe.

### Electrocorticography and Data Analysis

**FIGURE 2:**
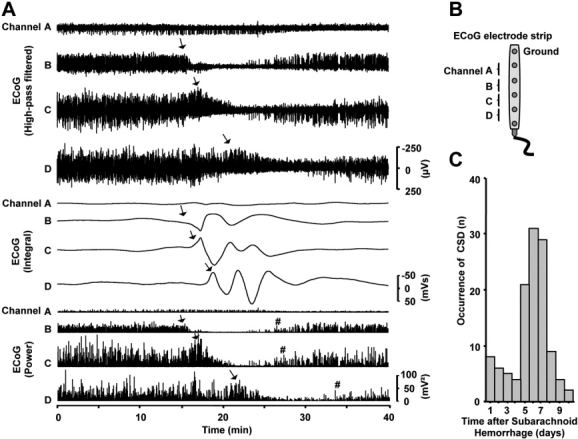
Subdural electrocorticography (ECoG). (A) A representative electrocorticogram 5 days after subarachnoid hemorrhage (SAH) over a time period of 40 minutes (Patient 2). The upper 4 traces (channels *A, B, C*, and *D*) demonstrate the high-passed filtered ECoG data. The ECoG displays a cortical spreading depression (CSD, *arrows*) propagating from channel *B* through *C* to *D*. The middle 4 traces show the integrals of each ECoG channel, with the corresponding integral changes indicated with arrows. The lowermost 4 show the power of ECoG and the rapid reduction of power due to the CSD. Time period from arrows to hash symbols represents the duration of the CSD^4^; channel *D* shows the longest duration of this typical CSD. Scales shown in channel *D* are also representative for the channels *A, B*, and *C*, respectively. (B) Schema of ECoG electrode strip: 5 bipolar electrodes generate the 4 channels: *A, B, C*, and *D*. The 6th electrode can be used as ground. (C) Histogram of CSD occurrence in SAH patients (n = 8; for day 1, n = 6) demonstrates that most CSDs (89 of 120 CSDs, ∼75%) occur between the 5th and 7th day after SAH.

The subdural ECoG was recorded and analyzed following a standardized COSBID protocol (see Supplementary Patients and Methods online for brief description).^4^,^9^,^10^ Figure 2A shows an original ECoG recording. We distinguished between single CSDs (interval between 2 CSDs ≥2 hours) and those that occurred repetitively within clusters with rather short intervals between consecutive CSDs (<2 hours) and with only small variations in interval length (<10%) in individual CSD clusters. We further differentiated between the 1st, 2nd, 3rd, and 4th or later CSD positions within the cluster.

### Brain Tissue Oxygen Partial Pressure Monitoring and Data Analyses

**FIGURE 3:**
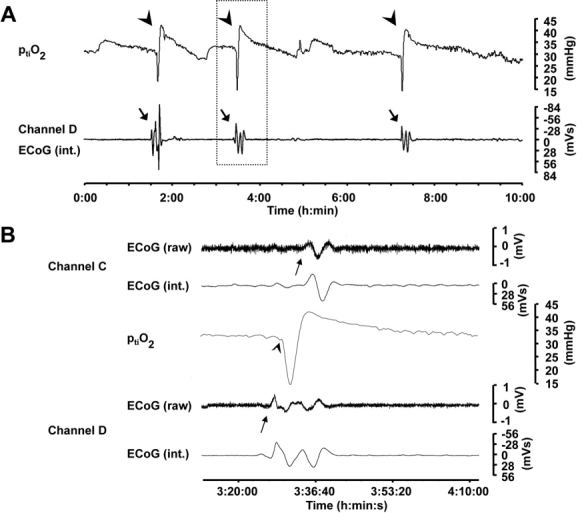
Coregistration of electrocorticography (ECoG) and tissue oxygen pressure (p_ti_O_2_). (A) A cluster of repetitive cortical spreading depolarizations (CSDs) in association with transient biphasic p_ti_O_2_ responses demonstrated in the coregistration of ECoG (*lower line*) and p_ti_O_2_ measurement (*upper line*) of a subarachnoid hemorrhage patient over a time period of 10 hours (Patient 5). Channel *D* (integral [int.]) is shown. The 3 CSDs are marked by the arrows; arrowheads demonstrate the corresponding biphasic p_ti_O_2_ responses. The long time period and the low baseline level of the ECoG integral reveal the spatial and temporal association with p_ti_O_2_ responses. (B) The enlargement of temporal resolution of a certain time period (*rectangle* in part A) and the addition of a second ECoG channel (*C*) (raw data and integral) reveal the exact CSD propagation (*arrows*) from channel *D* to *C* and the clear temporal relationship with the corresponding biphasic p_ti_O_2_ response (*arrowhead*).

p_ti_O_2_ was continuously quantified in the cortical tissue that was overlaid by the 6-contact-ECoG strip electrodes (compare Fig. 3). Because of the spatial distance between ECoG electrode and p_ti_O_2_ probe (≤8mm), p_ti_O_2_changes were accepted as coregistrations in a small range of ≤6 minutes around time points of CSD appearance. Such spatial distance may lead to a small time-displacement of the coregistration. We considered p_ti_O_2_ changes as significant p_ti_O_2_ responses if a rapid (≤ 120 seconds) p_ti_O_2_ deviation from the baseline (≥±2mmHg) was detected.

**FIGURE 4:**
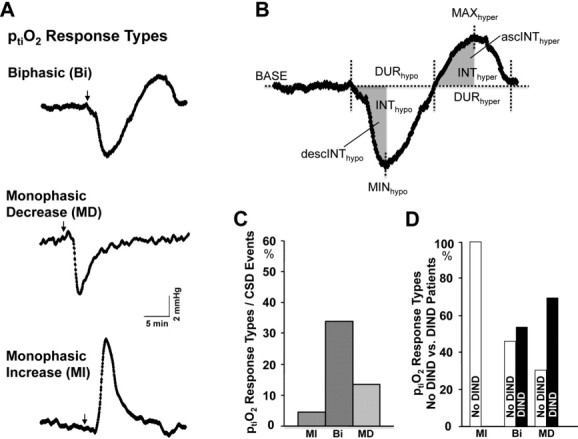
Three different types of tissue oxygen pressure (p_ti_O_2_) responses are found in the cortex of subarachnoid hemorrhage (SAH) patients in spatial and temporal association with cortical spreading depolarizations (CSDs). (A) Biphasic p_ti_O_2_ response with an initial decrease and a secondary increase, monophasic p_ti_O_2_ decrease, and monophasic p_ti_O_2_ increase. Arrows indicate the start of CSD in the electrocorticography (ECoG) channel next to the p_ti_O_2_ probe. (B) Magnification of the biphasic p_ti_O_2_ response of part A. Further detailed p_ti_O_2_ response analyses reveal values of specific features, integrals, and subareas of the p_ti_O_2_-curve: BASE = p_ti_O_2_ at baseline (30.60 [25.25, 36.70] mmHg), MIN_hypo_= the minimum (23.90 [16.75, 32.38] mmHg), DUR_hypo_= the duration (345 [207.5, 502.5] seconds), and INT_hypo_= the integral (12.76 [3.87, 18.94] arbitrary units [aU]) of the initial hypoxic phase. descINT_hypo_= (grey-shaded) integral representing the phase we designate “descent area,” that is, the area under the curve from start to minimum of the hypoxic phase. MAX_hyper_= the maximum (38.00 [28.30, 45.00] mmHg), DUR_hyper_= the duration (720.0 [540.0, 1090.0] seconds), and INT_hyper_= the integral (40.00 [12.50, 124.28] aU) of the secondary hyperoxic phase; ascINT_hyper_= second (grey-shaded) integral depicting an ascent area, that is, the area from start to maximum of the secondary hyperoxic phase. In some cases (compare with Fig 4A), p_ti_O_2_ shows an asymptotic return to p_ti_O_2_ baseline, which may distort curve analysis; thus the grey-shaded areas are additionally scrutinized to avoid this distortion. (C) Relative percentage frequencies of different p_ti_O_2_ responses to ECoG events (90 CSDs). Clear spatial and temporal associations of p_ti_O_2_ responses with EcoG events are found in 53.3 (42.7, 67.1)% of the CSDs (in total 47 of 90 CSDs, with association rates up to 90% in single individuals). The differentiation of all CSD-associated p_ti_O_2_ response types demonstrates that the biggest portion is contributed by biphasic p_ti_O_2_ responses (Bi); monophasic increases (MI) were rare in the severe SAH patients, and monophasic decreases (MD) were more frequent. (D) The 3 different p_ti_O_2_responses (compare with part C) in total ∼100% and their distribution of SAH patients with no ischemic neurological deficit (DIND) (*white bars*) versus SAH patients with DIND (*black bars*). All monophasic p_ti_O_2_ increases were found in SAH patients with no DIND and good outcome (extended Glasgow Outcome Scale ≥ 6). Biphasic p_ti_O_2_ responses were approximately similar in both patient groups, whereas monophasic decreases were typically found in patients developing DIND.

Because p_ti_O_2_is closely correlated with local CBF,^15^,^24^,^25^ we classified the p_ti_O_2_ alterations (Fig 4A) according to classifications used for CBF responses to CSDs: monophasic increases, biphasic alterations with an initial decrease and a secondary increase, and monophasic decreases.^19^ CSD-associated p_ti_O_2_ response analysis was also performed using the matching ECoG event criteria with differentiation between the 1st, 2nd, 3rd, and 4th or later temporal rank of the p_ti_O_2_ response within the cluster. Because not all SAH patients revealed clusters with such numerous CSD repetitions (≥4), in addition, we separately scrutinized patients with long clusters.

As elements of the CSD-associated p_ti_O_2_ response (see Fig 2B), we determined the preresponse p_ti_O_2_ baseline, the minimum and the duration of the p_ti_O_2_ decrease, and the maximum and the duration of the p_ti_O_2_ increase. We analyzed the curve integrals expressed as arbitrary units (aU) of hypoxic (INT_hypo_) and hyperoxic (INT_hyper_) p_ti_O_2_ responses, and assessed the portions from start at baseline to minimum as descent integral (descINT_hypo_) and to maximum as ascent integral (ascINT_hyper_), respectively. The latter parameter was chosen because the time needed for return to baseline was quite variable, in particular after reaching the secondary maxima.

### Digital Subtraction Angiography and Time-of-Flight Magnet Resonance Imaging

In 7 of 9 patients, we had the logistic possibilities to investigate proximal vasospasm by performing digital subtraction (DS) angiography or time-of-flight magnetic resonance imaging (MRI) on the 6th to 9th day after SAH.

### General Intensive Care Unit Monitoring

See Supplementary Patients and Methods online.

### Statistical Analysis

Results are expressed (if not otherwise stated) as median (1st, 3rd quartile); when necessary, ranges are also given. For nonparametric comparison between variables of ECoG and/or p_ti_O_2_ findings, the Friedman test of *k* dependent variables and/or the Wilcoxon test of 2 dependent variables were used. Correlation analyses were performed according to Spearman. *p* < 0.05 was chosen as the significance level. Statistical analyses were performed using SPSS for Windows (SPSS, Surrey, UK).

## Results

### Patients Characteristics

A summary of patients' clinical data is given in the Table. Two patients developed DIND, and 7 did not. All 7 patients undergoing DS angiography or time of flight MRI showed mild, moderate, or severe proximal vasospasm. In Figure 1, imaging findings of a patient developing DIND (Patient 1; see Fig 1A–J) are compared with those of a patient developing no delayed cortical infarcts (Patient 2; see Fig 1K–Q). Artifacts are seen, demonstrating the positions of subdural ECoG strip electrodes and adjacent p_ti_O_2_ probes. See also Supplementary Clinical Results online, especially for more detailed information on clinical courses illustrating the entirely different ECoG and p_ti_O_2_ findings, and outcomes of these 2 patients.

**TABLE:** Summary of Patients' Clinical Data

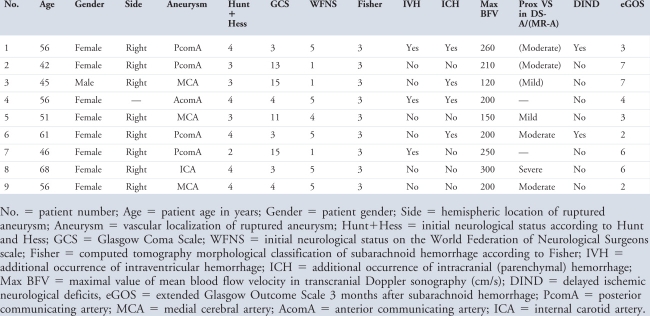

### Electrocorticogram

The total ECoG recording time in all patients amounted to 850 hours, with a median recording time of 144 (82, 184) hours in individual patients. We found a total of 120 CSD events in 8 of 9 patients (∼89%). Fifty-five of the 120 CSDs (∼46%) were found in the only 2 patients developing DIND, whereas a comparable number of only 65 CSDs (54%) were found in 7 SAH patients without delayed ischemia. In Patient 4, we were not able to detect CSDs, possibly because of a suboptimal strip electrode placement. Figure 2 shows an illustrative episode of a 4-channel (A-D) ECoG recording derived from bipolar recordings between successive neighboring electrodes 2 to 6 on the strip. The consecutive suppression in the high-pass filtered ECoG demonstrates the propagation of the CSD wave from channel B to D (arrows), whereas on channel A (recorded between electrodes 2 and 3), only minimal activity is left, indicating that electrodes 1 and perhaps 2 were positioned above already injured and therefore depolarized tissue. ECoG integral and power (see Fig 2A, lower panels) were calculated, to optimize the determination of starting and endpoints and thereby velocity and duration of CSD waves. The propagation velocity of CSDs along the electrode strip was 2.20 (1.34, 3.03) mm/min. CSD duration varied from 1 to 24 minutes, the median duration being 7:46 (4:31, 12:30) minutes:seconds. Approximately 75% of all CSDs occurred between the 5th and 7th day after SAH (see Fig 2C). We identified in individual patients a median number of 16 (9, 18) CSDs in total and of 5 (0, 7) CSDs per day, thus documenting that the repetition rate of CSDs was variable. Recurrent appearance of CSDs as temporal clusters was predominant (80 [48, 95]%), particularly in SAH patients developing DIND (89 [80, 98]%).

### Brain Tissue Partial Oxygen Pressure

During 90 of 120 CSD events, data were available from p_ti_O_2_ probes implanted adjacent to the electrode strips, yielding successful corecordings. In 53.3 (42.7, 67.1)% of the coregistrations in individual patients, we observed apparent spatial and temporal association between CSDs and p_ti_O_2_ responses, the highest association being 90% in 1 individual. Figure 3A shows illustrative CSD waves in an integrated ECoG channel (channel D) with simultaneously recorded biphasic p_ti_O_2_ changes, which were in close temporal association with the identified CSDs. The zoomed view (see Fig 3B, compare with rectangle in 3A) reveals that the p_ti_O_2_ response is preceded by the CSD wave on channel D, which then propagates to channel C. The pattern of p_ti_O_2_ responses in the examples given was biphasic, with a primary hypoxic phase followed by a secondary, longer lasting hyperoxic phase. Monophasic p_ti_O_2_ increases as well as decreases, however, were also observed. Figure 4A depicts examples of these 3 different types of p_ti_O_2_ responses. Figure 4B explains how we examined p_ti_O_2_ curves. The analysis revealed that most of the recorded p_ti_O_2_ responses were biphasic. Monophasic p_ti_O_2_ increases were found only in 2 individuals with good outcome (extended Glasgow Outcome Scale [eGOS] ≥6). Monophasic p_ti_O_2_ decreases were more frequent. Approximately 70% of these decreases were found in individuals who developed DIND in close temporal association with the occurrence of CSD clusters (see Fig. 4C,D).

### Influence of Number and Frequency of CSDs on CSD-Coupled p_ti_O_2_ Responses

In individual patients, both the absolute number and the number/day of CSDs were positively correlated with the duration of the hypoxic phase of biphasic CSD-associated p_ti_O_2_alterations (ρ = 0.430, *p* = .005; ρ = 0.444, *p* = .003, respectively), showing that primary hypoxia lasted longer, if CSDs became more frequent. Conversely, the absolute number of CSDs was negatively correlated with the duration of the secondary hyperoxic phase of the p_ti_O_2_ responses (ρ = −0.408, *p* = .008). Thus the secondary, possibly compensatory hyperoxygenation declined with increasing number of CSDs. Comparing primary hypoxia with secondary hyperoxia (INT_hypo_ respectively INT_hyper_) in patients with and without DIND, we found more pronounced hypoxic (19.90 vs 7.49 aU) and smaller or no hyperoxic responses (0.51 vs 41.49 aU) in DIND patients.

### P_ti_O_2_ Response Alteration in CSD Clusters

**FIGURE 5:**
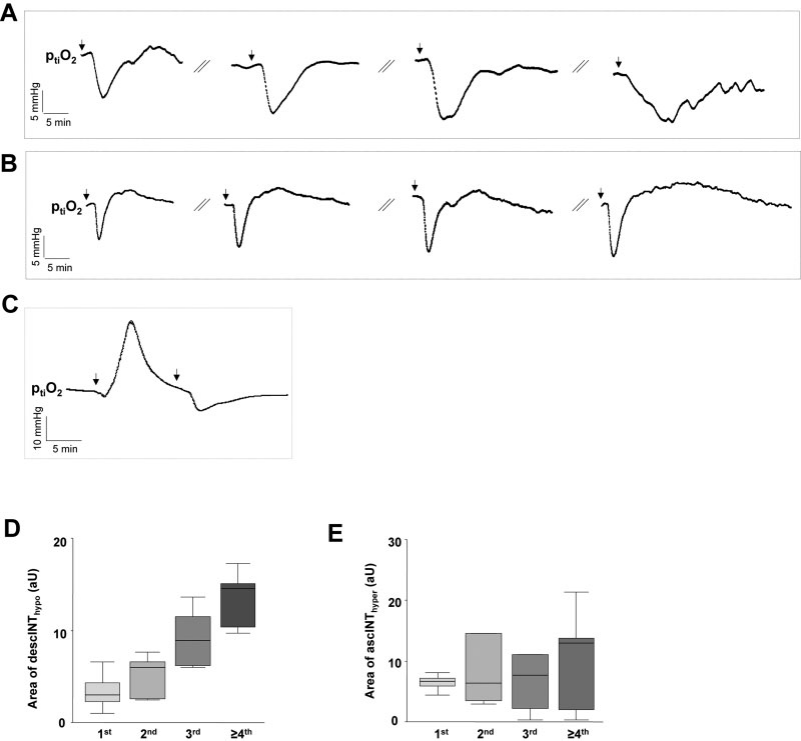
Cortical spreading depolarization (CSDs) within clusters and corresponding tissue oxygen pressure (p_ti_O_2_) responses. (A) An example (taken from Patient 1, 6 days after subarachnoid hemorrhage [SAH]) of 4 repetitive CSD-associated p_ti_O_2_ responses within a cluster (*arrows* in A–C represent start of CSD). The 1st p_ti_O_2_ response was biphasic, the 2nd and 3rd showed stepwise augmentation of primary hypoxic phases of the p_ti_O_2_ responses with subsequent small or almost nonexisting secondary hyperoxic phases, and the 4th showed a drastically enlarged hypoxic phase. Please note that particularly in the 4th p_ti_O_2_ response, the baseline level was not restored. This example shows an immediate transition from a biphasic to a monophasic decrease pattern of the p_ti_O_2_ response and may therefore reveal a change from normal to inverse neurovascular coupling and/or increased oxygen consumption, with the likelihood of secondary cortical ischemia after SAH. (B) The second example (taken from Patient 2, 7 days after SAH) of 4 repetitive CSD-associated p_ti_O_2_ responses within a cluster (*arrows*, see above). The primary hypoxic phases were stepwise slightly enlarged beginning with the 1st, then the 2nd, 3rd, and 4th response; however, a similar extension was seen in the secondary hyperoxic phases. In this example, all p_ti_O_2_ responses were biphasic in nature, and the p_ti_O_2_ baseline level was subsequently reestablished or even slightly exceeded, possibly representing normal (compensatory hyperemic) neurovascular coupling and/or simply transiently increased oxygen consumption not leading to ischemic transformation after SAH. (C) Examples of 2 CSD-associated p_ti_O_2_ responses with a rapid sequence of CSDs. Note that the period from 1st to 2nd CSD lasts only ∼12 minutes. The first p_ti_O_2_ response showed a biphasic pattern with a small hypoxic and a relatively large secondary hyperoxic phase, whereas the 2nd response revealed a deeper and prolonged hypoxic phase without a secondary hyperoxic phase, that is, a monophasic decrease of p_ti_O_2_. This example demonstrates a rapid transition from biphasic to monophasic decreasing p_ti_O_2_ pattern, perhaps due to the very small period between 2 repetitive CSDs. (D, E) Analysis of CSDs and corresponding p_ti_O_2_ responses within clusters. Box plots represent CSDs categorized by their specific order (1st, 2nd, 3rd, and ≥4th) showing median, quartiles, and full range of data. CSD-associated hypo- and hyperoxic p_ti_O_2_ response phases (areas of descINT_hypo_ or ascINT_hyper_, see Fig 4B) are shown. With ascending order of CSDs within the clusters (D) areas of the initial hypoxic phase increased significantly in a stepwise fashion within clusters (n = 5, *p* = 0.011, Friedman test). (E) Areas of the secondary hyperoxic phase showed no significant alterations (n = 5, not significant); however, CSDs ≥4th in a cluster suggested a trend to higher areas. aU = arbitrary units.

The high incidence of CSD clusters (96/120 CSDs) in general and particularly in DIND patients prompted us to analyze additionally the temporal alteration of p_ti_O_2_responses within clusters. These were defined by relatively short intervals between consecutive CSDs (<2 hours, mean ± standard deviation of all 96 CSDs within clusters: 44:14 ± 21:15 minutes:seconds) and by small variations in interval length (<10%). We analyzed clusters with ≥4 events obtained in 5/8 patients by categorizing events by their specific order as 1st, 2nd, 3rd, and 4th or higher rank within clusters to study CSD-associated p_ti_O_2_ responses in sequential order. Regarding biphasic p_ti_O_2_ responses in the examples shown (Fig 5A, B: Patients 1 and 2, see also Supplementary Clinical Results online), primary hypoxic decreases were enhanced within CSD clusters, whereas secondary hyperoxic increases appeared to be affected to a lesser extent. Overall, primary hypoxic phases of the respective p_ti_O_2_ responses (descINT_hypo_, see Fig 5D) were significantly augmented (n = 5, *p* = 0.011) within clusters, whereas secondary hyperoxic phases (ascINT_hyper_, see Fig 5E) did not show significant alterations. The ratio between primary hypoxic and secondary hyperoxic phases (analyzed as their respective subareas: descINT_hypo_/ascINT_hyper_) also showed significant increases in successive events within clusters (n = 5, *p* = 0.041). Considering all SAH patients with clusters (including those with only 2 successive events in some clusters), the comparison between hypoxic phases (descINT_hypo_) of the 1st versus the 2nd p_ti_O_2_ response showed a trend toward enhanced hypoxic responses of the 2nd response within clusters (n = 8, *p* = 0.091). Summarizing, prolonged clusters led to more pronounced and significant changes of p_ti_O_2_ responses.

Unlike in the examples with progressive, stepwise p_ti_O_2_ response modification, rapid, drastic alterations from a biphasic to a monophasic p_ti_O_2_ decrease pattern may occur, if a 2nd CSD quickly emerges before the previous p_ti_O_2_ response has returned to pre-event baseline. In such a case (Fig. 5C), the hypoxic phase increased considerably, and secondary hyperoxia was completely absent.

## Discussion

Our study provides a unique insight into disturbed oxygen availability to the SAH-injured human cerebral cortex in association with CSD occurrence. Initially, the cortex is not too seriously affected, but it may later develop cortical microcirculatory vasospasm and increased O_2_ consumption as responses to spontaneous CSD clusters. It would now appear that delayed ischemic neurological deterioration may result not only from major proximal arterial vasospasm, but in addition from CSD clusters that in particular contribute to delayed ischemia by causing subtle but noticeable stepwise alterations toward hypoxic tissue conditions. In consequence, CSD clusters may play a crucial role in the development of delayed ischemia after SAH,^4^,^15^,^26^ possibly in synergy with other mechanisms known to affect SAH-injured tissue, such as inflammation, blood–brain barrier disruption with edema formation and resulting mass effects, and endothelial dysfunction and thromboembolism.^1^,^27^,^28^ Thus the demonstration that CSD clusters occur and can reduce brain p_ti_O_2_may go some way toward explaining the variable association of delayed ischemia with proximal arterial vasospasm,^29^ and the existence of such a dual mechanism of delayed ischemia.^4^,^15^,^16^

We detected CSD-associated p_ti_O_2_alterations in >53% of CSDs despite an electrode to p_ti_O_2_probe distance of ≤8mm and despite the fact that the effective cortical area sampled by an ECoG electrode records amounts to ∼250mm^2^, whereas the p_ti_O_2_ probe analyzes only an area of ∼7.1 to 15mm^2^, comprising both cortical and subcortical tissue.^24^ In individual patients, we found an incidence of CSD–p_ti_O_2_ associations of up to 90%, demonstrating that CSD-associated p_ti_O_2_ changes are common and therefore a relevant pathophysiological factor in cortical tissue of SAH patients. Moreover, it gives rise to the assumption that consequences of CSD and associated microcirculatory disturbance^15^ may not be restricted to cortical tissue. Occasional lack of p_ti_O_2_ response association in individual patients with some successive CSDs may be explained by varying routes of CSD wave propagation,^9^,^10^,^19^ if the waves were for example induced at different sites and came into the rather large effective detection range of the ECoG electrode but not into the smaller range of the p_ti_O_2_ probe.

The propagation velocity of CSDs was comparable with Leão's classical description^5^ and recent clinical studies.^4^,^9^,^10^ The rate of CSD initiation peaked at days 5 to 7 after SAH (see Fig 2C), suggesting a possible role of CSD in DIND development, which has been reported to occur within approximately the same time frame.^1^,^23^ Other pathophysiological mechanisms develop earlier^1^,^30^ or at more or less unpredictable time points after SAH.^1^ Because 89% of CSDs arose within clusters in DIND patients, and because clusters altered p_ti_O_2_ responses in particular, we consider that these clusters may also play a role in DIND evolution. In contrast, single CSDs may be less harmful.^12^ The high incidence of CSD clusters in our severe SAH patients is comparable with other conditions requiring neurocritical care and exhibiting secondary ischemic deterioration, such as malignant hemispheric stroke.^10^

How might CSD clusters contribute to secondary injury progression after SAH? Experimental and clinical evidence demonstrates that CSD does not invariably induce compensatory vasodilatation (normal neurovascular coupling, as is known for physiological perfusion conditions), but can instead induce vasoconstriction (inverse coupling) if tissue conditions are unfavorable.^5^,^15^,^16^,^31^,^32^ In an SAH model,^16^ it was shown initially that inverse neurovascular coupling led to widespread focal infarcts. Inverse coupling was likewise found during hypoxia or systemic hypotension, and in the boundary zones of focal ischemia after middle cerebral artery occlusion.^18^–^20^ It is well known that p_ti_O_2_is mostly linked to regional CBF.^15^,^24^,^25^,^33^,^34^ The variable p_ti_O_2_responses found in our SAH patients (see Fig 2A, C) resemble the patterns of CBF responses coupled to CSD/PID described in animals.^16^,^19^,^20^ Most of the p_ti_O_2_responses examined were biphasic in nature (see Fig 2C), resembling biphasic CBF response patterns in boundary zones of ischemic foci^19^,^32^,^35^; hence, we infer that our recordings were performed largely in metabolically disturbed but not yet severely injured tissue compartments. Monophasic hypoxic episodes indicate altered or missing secondary, compensatory hyperoxic phases, worsening in vascular reactivity, and thus deterioration of tissue conditions.

It seems, therefore, that the complex antagonism between vasoconstrictive and vasodilator mechanisms after SAH^36^ is progressively disturbed during ongoing CSD activity. Various intra- and extracellular mechanisms influencing coupled responses have been reported, some leading to intracellular calcium ([Ca^2+^]_i_) accumulation in smooth muscle cells.^36^,^37^ Repetitive CSDs may support this [Ca^2+^]_i_ accumulation and promote microvascular spasm and NO resistance.^38^ Other putative mechanisms may include activation of matrix metalloproteinase-9 followed by dysfunction of the neurovascular unit.^39^ As a mechanistic explanation for DIND, CSI has recently attracted interest.^1^,^4^,^11^,^40^ It has been shown that in the presence of elevated extracellular potassium concentration ([K^+^]_e_), depletion of NO may alter the vasomotor response to CSD from vasodilatation to vasoconstriction, resulting in ischemic transformation.^16^,^32^,^35^ Elevated [K^+^]_e_ generally characterizes any state of energy shortage. The vasoconstrictor capacity of [K^+^]_e_ easily dominates SAH tissue, because NO is trapped by the potent NO scavenger hemoglobin, both hemoglobin and K^+^ being released from erythrocytes in the subarachnoid space after SAH. Vasoconstriction then worsens energy delivery to this tissue, and in turn, K^+^ release is enhanced, creating a vicious cycle that inhibits repolarization and augments ischemia. CSD clusters with repetitive neuronal and astrocytic de- and repolarizations will reinforce such a cycle, as does repetitive vasoconstrictive activation of smooth muscle cells, which also utilize adenosine triphosphate (ATP) and O_2_. Accordingly, an altered metabolic state has been described for perivascular sites during CSD.^41^ Smooth muscle relaxation, as a component of vasodilatation, also requires ATP (flexibilizer function of ATP). In the case of repetitive CSDs with ATP depletion due to ATP-depended vascular responses^18^,^19^ and multiple neuronal and astrocytic repolarizations,^7^–^9^ the relaxation component of vasodilatation may therefore be negatively influenced. Furthermore, adenosine, a potential vasodilator and a metabolite of ATP degradation present in metabolically disturbed tissue, failed to improve vasodilatation after CSDs.^32^ Hence, CSD clusters may provoke repetitive vasoconstriction^19^ with gradually decreasing likelihood of vasodilatation, and progressively modified p_ti_O_2_ responses may finally result in cortical microvasospasm. Delayed laminar infarction in cortex, as found typically in SAH patients, supports this interpretation.^38^ Because in our study hypoxic portions of the CSD-associated p_ti_O_2_ response increased, and hyperoxic portions decreased or were completely absent after recurrent CSDs (see Fig 5), we assume that a rapid sequence of CSDs generates a vulnerable phase that potentiates hypoxic tissue conditions. Moreover, further studies are necessary to understand the complexity of delayed ischemia after SAH and the role of CSD clusters for this complication. The likely role of clusters in degrading tissue of marginal viability is illustrated by the detection with rapid sampling microdialysis of stepwise depletion of cortical tissue glucose during a cluster of CSDs.^42^

P_ti_O_2_ recordings inevitably reflect both delivery and consumption of tissue oxygen.^24^,^34^ CBF alterations linked to CSD have recently been shown to correlate with p_ti_O_2_ changes.^15^ CSD—more specifically, repolarization of neuronal membrane potentials—markedly increases energy metabolism^7^–^9^,^13^ and depletes brain glucose.^40^,^42^ Complete loss of compensatory vasoreactive power would result in sustained reduction of CBF and/or p_ti_O_2_ to zero,^19^ reflecting spreading ischemia.^15^,^16^,^19^ We were not able to observe such a phenomenon by p_ti_O_2_ monitoring in our study, presumably because only by chance, recordings with strip electrodes positioned at only 1 site in the surrounding of SAH would pick up the phenomenon. Retrospective analysis of imaging results revealed that p_ti_O_2_ probes were never positioned in tissue compartments undergoing delayed ischemia, offering an explanation for the lack of sustained p_ti_O_2_ reduction. Responses with long monophasic p_ti_O_2_ reductions found particularly in DIND patients may be interpreted as a preliminary stage of such delayed, terminal transformation as seen in spreading ischemia.^15^,^16^,^19^,^35^ It is obvious that recordings at only 1 location are suboptimal regarding a comprehensive analysis of the surroundings of injuries, and multiple site p_ti_O_2_ measurements would therefore be favorable but difficult to perform. A further limitation of the study is the lack of adequate and frequent imaging techniques like MRI (perfusion-weighted imaging, diffusion-weighted imaging) for the possible detection of spreading ischemia as shown in our examples. Logistic reasons and intensive care made this management impossible in some patients of our study. The COSBID plans a multicenter study that aims at following such sequential imaging protocol in all patients. In this study, we also aim to test intrinsic neurovascular function and the capacity for local cerebrovascular autoregulation and oxygen reactivity,^1^ because these factors may be of relevance for the biphasic p_ti_O_2_ cycle.

In conclusion, CSDs may impair microvascular function and O_2_ availability, likely with increased O_2_ consumption, in human cerebral cortex after SAH, especially if they occur in clusters. Our results indicate that this is related to secondary hypoxic transformation and the likelihood of DIND. The correspondence of experimental and clinical data suggests that we can now cautiously translate the well-known impact of depolarizations on pathophysiological outcome in experimental models into the clinical situation. Considering the importance for outcome of secondary deterioration in SAH, CSD may represent a promising target for therapeutic intervention to improve poor outcome and mortality after SAH.

## Authorship

B.B. and R.G. contributed equally to this work.

## Potential Conflicts of Interest

Anthony Strong was paid a consultancy fee, as well as receiving payment for development of educational presentations by Codman Johnson & Johnson.
