# Supplementary material for: Recurrent spreading depolarizations after subarachnoid hemorrhage decreases oxygen availability in human cerebral cortex
Source: Ann Neurol. 2010 May;67(5):607–17. doi: 10.1002/ana.21943 (PMC2883076; doi:10.1002/ana.21943)
Supplement: Supplementary file 1 [file ana0067-0607-SD1.doc]

**Supplementary Clinical Results** **(online)**

***Patients and their Clinical Characteristics (online)***

Patients were 56 (quartiles 45.5, 58.5) years old (range 42-68) and predominantly female (one male patient). In most patients, the right hemisphere was affected (one case with AcomA aneurysm with pronounced left hemispheric SAH). All patients suffered from massive SAH caused by spontaneously ruptured aneurysms of different cerebral arteries resulting in poor initial cranial CT findings and accordingly Fisher grade^21^ 3, with additional ventricular and/or intraparenchymal hemorrhage in three respectively four individuals. Hunt and Hess grade^22^ was 3 or 4, with one exception (grade 2). Scores for Glasgow Coma Scale (GCS) and World Federation of Neurological Surgeons Scale (WFNC) were 4 (3, 13) and 5 (1, 5), respectively. The maximal mean blood flow velocity was 200 (175, 255) cm/s (see table 1). DIND was found in two individuals with corresponding low extended Glasgow outcome scale (eGOS)^23^ value ≤3 after three months.

**Two individual clinical courses after SAH**

*Patient 1:*

A 56-year old female presented with Hunt and Hess grade 4 and Fisher grade 3 SAH. Clinical characteristics are summarized in Tab. 1, and a selection of imaging findings is shown in Fig. 1 (upper panel). Initially unconscious, she suffered from widespread basal SAH with right-sided sylvian and intraparenchymal involvement. Sedation was used, and an external ventricular drain was temporarily established. Digital subtraction angiography (DSA) showed an aneurysm located at the posterior communicating artery (PcomA) as source of SAH; the aneurysm was coiled one day after SAH. At this stage no vasospasm was seen, but changes in the ICH border zone were associated with mass effects. On temporary reduction of sedation, patient did not regain full consciousness, but moved all limbs to pain. However, decompressive hemicraniectomy was necessary two days after SAH on account of right hemispheric brain swelling with ICP rise; during surgery the ECoG strip electrode was placed, with an adjacent p_ti_O_2_ probe (Fig. 1A). At this time, CT did not show any cortical infarcts (Fig. 1B-D). ECoG showed a first single CSD on the fourth day after SAH, associated with a biphasic p_ti_O_2_ alteration (initial p_ti_O_2_ decrease followed by a secondary increase). On the same day, perfusion (PW) and diffusion weighted (DW) MRI showed no signs of hypoperfusion and cortical ischemia (Fig. 1E, F); the detailed temporal course of BFV (measured by TCD in the right/left MCA on the 2^nd^-4^th^-6^th^-8^th^ & 10^th^ day after SAH) was 70/60-85/80-160/125-220/135-260/150 cm/s, respectively. On the fifth day, and also on the following 2 days, multiple CSD clusters with CSD-associated biphasic p_ti_O_2_ alteration were observed. The p_ti_O_2_ response pattern changed progressively from biphasic to monophasic, and the hypoxic phase of the response was stepwise augmented in severity and duration (Fig. 5A). Due to sedation, we were not able to observe clinical (DIND) manifestations of these CSD clusters as was previously reported^4^. However, perfusion and diffusion weighted MRI (Fig. 1G,H) and subsequent T1 and T2 MRI (Fig. 1I,J) showed hypoperfusion, edema formation and development of multiple cortical infarcts after occurrence of the CSD cluster, with a monophasic (hypoxic) p_ti_O_2_ response pattern presumably indicating inverse neurovascular coupling (and/or increased metabolic load) with consecutive ischemia^1,4,16^. These findings were linked to DIND (seen after withdrawal of sedation) with delayed neurological impairments at discharge from ICU and poor outcome three months after SAH (eGOS 3).

It should be mentioned that mass effects of ICH in particular might additionally promote the poor outcome of this individual patient.

*Patient 2:*

A 42-year old female SAH patient presented with Hunt and Hess grade 3 and Fisher grade 3 (see also Tab. 1 and lower panel of Fig. 1 respectively for more detailed clinical and imaging characteristics). The patient was transferred to neurosurgical care one day after sudden onset of neck- and headache, followed by severe vomiting. Additionally, there were neuropsychological deficits and a mild sensorimotor deficit on the left. CT with CT-angiography indicated an aneurysmal SAH (tentative diagnosis of a symptomatic PComA aneurysm) one day after SAH. An external ventricular drain was temporarily established because of deranged CSF circulation. DSA confirmed the rupture of a right PcomA aneurysm ; no signs of proximal or distal vasospasm were seen at this time point. Because of the broad-necked configuration of the aneurysm, occlusion by coiling was not indicated. The aneurysm was clipped surgically two days after SAH, and concomitantly, an ECoG strip electrode and an adjacent p_ti_O_2_ probe were implanted (Fig. 1K-Q). No signs of cortical ischemia were seen at this time on CT. ECoG and p_ti_O_2_ monitoring revealed a single CSD on the 3^rd^ day after SAH, no CSDs on the 4^th^ day, and a cluster of only two CSDs followed by three more single CSDs on the 5^th^ and one on the 6^th^ day. On the 7^th^ day, two clusters with 4 respectively 3 CSDs and 2 single CSDs were observed. P_ti_O_2_ responses showed slightly augmented primary hypoxic phases but also enlarged hyperoxic phases within CSD clusters (Fig. 5B) documenting a persistent biphasic p_ti_O_2_ pattern. Moreover, it should be noted that p_ti_O_2_ baseline was somewhat increased subsequent to clusters, and after long CSD-free intervals, p_ti_O_2_ responses reverted to biphasic patterns similar to those observed previous to clusters, indicating “normal” neurovascular coupling. Consistent with this finding, MRI imaging showed in this patient no signs of perfusion deficit (Fig. 1N) and cortical ischemia at the 8^th^ day after SAH, neither on DWI (Fig. 1O) nor on T1 and T2 images (Fig. 1P,Q). BFV of the right MCA peaked on this day with 210 cm/s (BFV course in the right/left MCA was 80/80-135/105-205/95-210/125-180/180 cm/s, respectively). Further CT imaging confirmed this positive outcome, as did the patient’s good clinical recovery, with rapid discharge from the ICU and favorable outcome (eGOS 7) three months after SAH.
