# Supplementary material for: Recurrent spreading depolarizations after subarachnoid hemorrhage decreases oxygen availability in human cerebral cortex
Source: Ann Neurol. 2010 May;67(5):607–17. doi: 10.1002/ana.21943 (PMC2883076; doi:10.1002/ana.21943)
Supplement: Supplementary file 2 [file ana0067-0607-SD2.doc]

**Supplementary Patients and Methods (online)**

**Electrocorticography and Data Analysis**

In brief, a commercial ECoG strip comprising 6 platinum electrodes with an electrode centre to centre distance of 10 mm was used. The most remote electrode or an external skin-electrode was used as ground. The other electrodes continuously acquired four bipolar active data channels (A, B, C and D). For pre-amplification we used two 2-channel amplifiers (Dual Bioamp, ADInstruments, New South Wales, Australia). The lower frequency limit of the alternating current (AC) amplifiers was 0.02 Hz (29% attenuation) [0.002 Hz (90% attenuation)]. The ECoG data were recorded, digitized and logged (Powerlab 16SP with Chart software, ADInstruments) for periods of up to ten days. The transient ECoG amplitude reduction was examined and classified as previously described^4,9,10^.

To amplify transient periods of ECoG amplitude reduction and to reverse the derivative transformation caused by the AC-filtration, the time integral of the ECoG signal was calculated, followed by subtraction of the general slope of the integral. This procedure allowed the visualization of slow potential changes (SPC), which developed approximately synchronously with the CSD. Furthermore, the SPC had opposite phases in adjacent channels leading to clear assignment of each component of the SPC to a single electrode demonstrating the active location of SPC.

The ECoG signal (AC) variations were filtered (0.5-70 Hz pass) and squared to obtain the power in the normal EEG-frequency bands. Moreover, the integral of the power of the ECoG signal was calculated to afford online monitoring by better visualizing the CSD on the ICU. The maximal duration of suppressed ECoG activity was used as an indirect index of the brain’s tissue energy status since restoration of ECoG activity after spreading depolarization is energy dependent^4,7-9,12,13^. This duration of ECoG depression until recovery was measured as the interval between depression onset and onset of restoration of activity using the integral of power of the high-pass filtered activity (lower frequency limit, 0.5 Hz; time constant decay, 60s), as previously reported^4^. The time points of each ECoG channel representing a depression were defined when at least two channels showed evidence of a CSD according to the standardized analysis^4,9,10^,.

**General ICU Monitoring**

Capillary blood oxygen saturation (SaO_2_), respiration rate and arterial blood pressure were measured continuously (SC 9000, Siemens Erlangen, Germany). The intracranial pressure was measured and maintained below 20-25mmHg in comatose (GCS≤8) and/or sedated patients. Arterial blood oxygen partial pressure, arterial carbon dioxide partial pressure, hemoglobin, electrolytes and plasma glucose were measured every 60-120min. Plasma glucose was kept in the range of 4.4-8.2mM (80-150mg/dl) using insulin if necessary. All data were collected using a data acquisition computer system. Blood flow velocity (BFG) in the proximal cerebral arteries was measured every 24 hours by transcranial Doppler sonography (TCD). Brain imaging (CT or, less often, MRI) were performed on admission, after surgical or interventional treatment and/or on 2^nd^, 5^th^ and 14^th^ (±1) day after SAH. In rare cases, the clinical situation led to further imaging. Additional MRI was performed on 6-9^th^ day after SAH (not Mannheim, for logistic reasons). In some patients, we performed perfusion MRI (“time to peak”: A delay of perfusion ≥+4 sec. was defined as critically hypoperfused.) DIND and delayed cortical infarction were defined as previously reported^4^.
